# Adolescents’ Responses to Peer Disclosure of Teen Dating Violence: Relationship Configuration, Response Intentions, and Protective Adult Support

**DOI:** 10.3390/bs16071043

**Published:** 2026-06-23

**Authors:** Francesco Sulla, Andreana Lavanga, Margherita Santamato, Nunzia Merafina, Salvatore Adam Leone, Giulia Fiorentino, Anna Sorrentino

**Affiliations:** 1 Department of Human Studies, University of Foggia, 71121 Foggia, Italy; andreana.lavanga@unifg.it (A.L.); nunzia.merafina@unifg.it (N.M.); salvatore_leone.589434@unifg.it (S.A.L.); giulia.fiorentino@unifg.it (G.F.); 2 Department of Psychology, University of Campania ‘Luigi Vanvitelli’, 81100 Caserta, Italy; margherita.santamato@unicampania.it (M.S.); anna.sorrentino1@unicampania.it (A.S.); 3 Department of Research and Humanities Innovation, University of Bari ‘Aldo Moro’, 70122 Bari, Italy

**Keywords:** teen dating violence, adolescents, peer disclosure, response intentions, help-seeking, vignette methodology, supportive adult relationships

## Abstract

Teen dating violence (TDV) is a relevant form of adolescent interpersonal violence, yet little is known about how adolescents intend to respond when a peer discloses victimization and whether these responses facilitate access to supportive adults and other protective resources. This study examined adolescents’ intended responses following peer disclosure of TDV using a vignette-based design that extended prior work by including four relationship configurations: heterosexual male perpetrator/female victim, heterosexual female perpetrator/male victim, male same-sex couple, and female same-sex couple. Participants were 655 adolescents aged 12 to 18 years from secondary schools in Southern Italy. Descriptive findings showed that supportive and relational responses were most frequently endorsed, including listening to the friend, helping them decide what to do, reassuring them, and encouraging them to talk to trusted others, whereas institutional responses were endorsed less often. Stratified chi-square analyses indicated that condition effects were selective rather than pervasive and were concentrated mainly in responses involving escalation to adults or authorities. Across subgroups, the heterosexual female-perpetrator/male-victim condition was most consistently associated with lower intervention-oriented responding and/or greater uncertainty, whereas the heterosexual male-perpetrator/female-victim condition more often elicited active intervention. The findings suggest that adolescents’ responses to peer disclosures of violence are shaped not only by prosocial intentions but also by the social recognizability of the violent scenario, with implications for validation, access to supportive adults, and inclusive school-based prevention.

## 1. Introduction

Teen dating violence (TDV) is a significant public health concern during adolescence and is associated with a broad range of adverse outcomes, including depression, anxiety, suicidal ideation, risky sexual behavior, school maladjustment, reduced well-being, and later revictimization in intimate relationships (Piolanti et al., 2023; Sánchez-Zafra et al., 2024). Although prevalence estimates vary depending on the type of violence assessed and the measures adopted, the available literature consistently indicates that TDV is far from rare. A European systematic review highlighted substantial prevalence rates for both perpetration and victimization, while also underscoring marked heterogeneity in measurement practices and a relative scarcity of European studies compared with the North American literature (Tomaszewska & Schuster, 2021). Within the Italian context, evidence is even more limited. In particular, Sorrentino et al. (2023) noted that few studies in Europe and Italy have examined TDV risk factors, and their longitudinal study with early adolescents from Southern Italy represents a particularly important point of reference because it situates TDV within the developmental ecology of aggressive and antisocial behaviors in this specific territorial context.

A robust finding across the help-seeking literature is that adolescents involved in dating violence tend to turn first to informal sources of support, especially friends and family members, rather than to professionals or formal services. Ashley and Foshee (2005) found that most adolescent victims and perpetrators did not seek help for dating violence at all, and that those who did primarily relied on friends and family. Similarly, Fernet et al. (2019) showed that adolescents and emerging adults often perceived friends and parents as more accessible than professionals, whereas formal help-seeking was hindered by shame, privacy concerns, uncertainty about the seriousness of the experience, and reluctance to disclose intimate details to strangers. These findings suggest that peer contexts are central to adolescents’ responses to dating violence, yet the way peers themselves interpret and respond to such disclosures remains underexamined, despite the fact that these responses may shape whether victims feel validated, supported, and safe enough to seek further help, with potentially important consequences for psychological adjustment. Qualitative evidence further suggests that informal help-seeking in the context of romantic difficulties and dating violence is not a single act, but a process involving problem recognition, the decision to seek help, and the selection of a support source, with peers often functioning as first responders in this trajectory (Fernet et al., 2021).

More broadly, adolescent help-seeking after interpersonal victimization depends not only on willingness to disclose, but also on problem recognition, perceived severity, expectations about others’ reactions, and the perceived accessibility of support figures. Research on adolescent bystander processes suggests that intervening is shaped by a combination of situational appraisal, perceived norms, responsibility, and anticipated social consequences (Casey et al., 2017). In addition, quantitative evidence indicates that adolescents’ expected bystander intervention in TDV is associated with factors such as empathy, acceptance of violence, and positive student–teacher relationships (Cerda-Smith et al., 2022). Together, these findings support viewing peer disclosure not as an isolated event, but as an early decision point in a broader help-seeking and support-mobilization process.

This issue is also relevant from a school mental health perspective. Research on students’ perceptions of teachers’ responses to bullying shows that active and supportive responses are associated with stronger reporting, school belonging, perceived fairness, and academic adjustment, whereas passivity, inconsistency, or contradictory reactions may undermine safety and trust in school settings (Muñoz-Fernández et al., 2026). This broader school-based perspective is also consistent with research indicating that many adolescents have opportunities to act as bystanders in situations involving dating or sexual violence, but that intervention depends in part on whether they feel able to recognize the situation as serious and to respond effectively (Cerda-Smith et al., 2022). Relatedly, because adolescents tend to rely heavily on peers when facing relationship difficulties, understanding their perceived capacity to respond when witnessing TDV or receiving a friend’s disclosure is particularly important for prevention and early support (Kan et al., 2022; Van Camp et al., 2014). Although TDV differs from bullying in important ways, both involve adolescent aggression disclosed, interpreted, and managed within peer and school ecologies; accordingly, adolescents’ coping intentions following TDV disclosure may be consequential not only for immediate support but also for later psychological adjustment (Fernet et al., 2019; Matuschka et al., 2022). In addition, adolescents do not seek informal support only to “report” violence in a narrow sense; they may seek peers and family members to obtain advice, express emotions, gain an external perspective, be listened to and comforted, and receive validation for their interpretation of the situation (Fernet et al., 2021).

This gap was recently addressed by Morrison et al. (2023), who used vignette methodology to examine adolescents’ perceptions of dating violence and their intended responses to peer disclosure. Their findings showed that the type of violence, along with participants’ age and gender, influenced blame attributions, interpretations of violence, and response intentions. However, Morrison et al. focused exclusively on heterosexual scenarios involving a male perpetrator and a female victim and explicitly noted the importance of extending this line of research to other relationship configurations. This limitation is theoretically important because some forms of dating violence may be more socially recognizable than others and may therefore more readily activate scripts of concern, seriousness, intervention, or minimization; in turn, these interpretive differences may affect whether adolescents mobilize supportive responses that facilitate disclosure, safety, and access to protective resources, or instead react with uncertainty and inaction (Morrison et al., 2023). This point is consistent with qualitative findings showing that many adolescents struggle to define coercion, jealousy, and controlling behaviors as abusive, at times interpreting them instead as signs of love or relational involvement (Fernet et al., 2021).

The need to move beyond a single heterosexual configuration is further supported by recent evidence on sexual and gender minority youth, including adolescents whose sexual orientation and/or gender identity or expression falls outside cisgender heterosexual norms (e.g., lesbian, gay, bisexual, transgender, non-binary, and other gender-diverse youth). In their systematic review, Sulla et al. (2025) showed that sexual and gender minority youth face elevated vulnerability to TDV due to minority stress and systemic inequalities, with risk factors spanning demographic and identity-related, psychological, violence-related, relational, family, school, and community domains. Importantly, the review emphasizes that the current literature often conflates sexual orientation and gender identity, under-examines subgroup-specific experiences, and rarely captures forms of violence that are especially salient in sexual and gender minority relationships, such as identity-based abuse, outing, or other dynamics linked to stigma and non-inclusive environments. The same review also notes that some studies suggest that same-gender couples may report higher rates of bidirectional violence than heterosexual couples, while most TDV research continues to focus narrowly on more traditional victimization frameworks (Sulla et al., 2025). Taken together, these points provide a strong rationale for expanding vignette-based TDV research beyond canonical heterosexual dyads and for including same-sex relationship conditions in the present design.

A further reason to broaden this line of inquiry comes from recent work on protective factors in adolescent dating violence, as well as from research showing that students’ perceptions of adults’ responses to peer aggression carry implications for belonging, safety, and adjustment in school contexts. A recent socio-ecological systematic review (Humaira et al., 2026) concluded that the literature has focused predominantly on risk factors, whereas protective processes remain comparatively underdeveloped as an object of study. Across the reviewed studies, the most consistent protective factors were social support from peers, family, and teachers, followed by school-based interventions, emotional intelligence, meaningful communication with parents, and social norms that support collective action against violence. Importantly, this review argues that resilience to adolescent dating violence is multidimensional and emerges from the interaction between individual capacities and the social environment, rather than from individual characteristics alone (Humaira et al., 2026). This view is consistent with recent evidence indicating that students interpret adult responses to peer aggression as socially meaningful cues that shape fairness, safety, school belonging, and willingness to seek help (Muñoz-Fernández et al., 2026). This perspective is particularly useful for the present study because adolescents’ responses to a peer’s disclosure can be conceptualized as one concrete expression of the protective social ecology surrounding TDV: supportive peer responses may encourage validation, disclosure, and access to help, whereas uncertainty, minimization, or disengagement may contribute to continued isolation and psychological distress (Fernet et al., 2021; Muñoz-Fernández et al., 2026). Importantly, informal support appears to be most accessible when it is embedded in relationships characterized by trust, reciprocity, relevant experience, and a respectful, non-judgmental climate. From this perspective, adolescents’ responses to peer disclosure may be understood not only as response intentions, but also as potential gateways to supportive adult relationships and other external protective resources (Nappa et al., 2021; Fernet et al., 2021).

Furthermore, recent school-based evidence suggests that the availability of adult support cannot be taken for granted. In upper secondary schools, teachers tend to report greater engagement in promoting gender equity and relationship-relevant education than students perceive, through both the hidden and the explicit curriculum. This points to a gap in the visibility and recognizability of adult practices. This issue is relevant because adolescents may be less likely to activate school- or adult-oriented coping responses when such resources are not experienced as clearly accessible, coherent, and credible in everyday school life (Lavanga et al., 2026).

### The Current Study

The present study builds on Morrison et al. (2023) by using vignette-based methodology to investigate adolescents’ response intentions to peer disclosures of TDV while extending the relational configurations represented in the scenarios. Specifically, unlike Morrison et al., the present study included four conditions: a heterosexual male-perpetrator/female-victim condition, a heterosexual female-perpetrator/male-victim condition, a male same-sex condition, and a female same-sex condition. In doing so, the study addresses both a contextual gap—given the still limited Italian literature on adolescent dating aggression and TDV, including evidence on prevalence and gender/age differences (Cuccì et al., 2020) and on risk processes in Southern Italy (Sorrentino et al., 2023)—and a conceptual gap, in light of the growing evidence on TDV among sexual and gender minority youth (Sulla et al., 2025).

The study addressed three main questions. First, which response options are most frequently endorsed following a peer disclosure of TDV? Second, do adolescents’ intended responses vary across relationship configurations, particularly for responses involving escalation to adults or authorities? Third, does the association between vignette condition and response category vary as a function of sex assigned at birth, after adjusting for age?

Based on prior research, we expected supportive and relational responses to be endorsed more frequently than formal or institutional ones. We further expected condition effects to emerge especially for responses involving escalation to adults or authorities, because these responses are likely to be particularly consequential for validation, safety, and access to protective support. Finally, given prior findings suggesting that adolescents’ responses to violence may vary by age, sex, empathy, and school relational context (Cerda-Smith et al., 2022; Morrison et al., 2023; Van Camp et al., 2014), we examined whether vignette condition and sex assigned at birth interacted in predicting response categories.

## 2. Materials and Methods

### 2.1. Participants

Participants were 655 adolescents aged 12 to 18 years (M = 14.64, SD = 1.52) recruited from lower- and upper-secondary schools in Southern Italy. The sample comprised 372 participants assigned female at birth (56.8%) and 283 assigned male at birth (43.2%). Participants attended schools in the provinces of Foggia (n = 459, 70.1%) and Barletta-Andria-Trani (BAT; n = 21, 3.2%) in Apulia, and Avellino (n = 126, 19.2%) and Caserta (n = 49, 7.5%) in Campania. In terms of school grade, 100 students (15.3%) were enrolled in the second year of lower-secondary school, 93 (14.2%) in the third year of lower-secondary school, 162 (24.7%) in the first year of upper-secondary school, 151 (23.1%) in the second year of upper-secondary school, and 149 (22.7%) in the third year of upper-secondary school.

### 2.2. Measures

Sociodemographic information included age, sex assigned at birth, and school grade. Participants were randomly assigned to one of four vignette conditions: (1) a heterosexual male-perpetrator/female-victim condition (n = 166, 25.3%), (2) a heterosexual female-perpetrator/male-victim condition (n = 170, 26.0%), (3) a male same-sex male-perpetrator/male-victim condition (n = 154, 23.5%), and (4) a female same-sex female-perpetrator/female-victim condition (n = 165, 25.2%). Each condition included three hypothetical TDV episodes involving two adolescents and depicting verbal/emotional TDV, relational TDV involving control and threatening behaviors, and physical TDV. The term dating violence was not explicitly mentioned. The vignettes (see Supplementary Materials for the full text; Text S1) were designed to be comparable in length and as similar as possible in structure, with the same introduction, contextual framing, and conclusion across conditions, varying only in the gender configuration of the victim–perpetrator dyad. This structure was inspired by Morrison et al. (2023), who likewise used hypothetical scenarios, avoided explicitly labeling the events as dating violence, and kept the vignettes highly comparable in format and length across conditions. After reading the three episodes within their assigned condition, participants completed a brief 14-item survey assessing possible peer responses to the disclosed situation overall.

Peer response items. Participants completed 14 adapted response items (see Supplementary Materials; Text S2) derived from the response-to-disclosure framework used by Morrison et al. (2023). The items assessed whether participants would endorse a range of possible responses to a peer disclosure of TDV, including supportive, peer-oriented, and adult-/institution-oriented actions. Response options were “I do not know”, “Yes”, and “No”. For the primary inferential analyses, these response categories were treated as nominal rather than ordinal. Although “Yes” and “No” represent opposite endorsement positions, “I do not know” was conceptualized as a qualitatively distinct uncertainty response rather than as an intermediate point on a linear continuum; accordingly, no equal-spacing or proportional-odds assumption was imposed. Following Morrison et al. (2023), the adapted items were not assumed a priori to constitute a single homogeneous scale. Instead, they were treated as a heterogeneous set of possible peer responses to disclosure, and their internal organization was examined empirically through inter-item associations and exploratory dimensionality analyses.

In the present study, informal responses refer to actions that remain within peer or family networks rather than involving formal institutions; relational responses refer to listening, reassurance, and supportive discussion; and accompaniment-based responses refer to helping the friend decide what to do or encouraging them to seek further support without taking control away from them.

### 2.3. Procedure

Schools were recruited after invitation letters had been sent in September 2024 to school principals in the areas served by the two universities with which the authors are affiliated, following a presentation of the study to representatives of the Regional School Offices. Data collection took place between October and December 2024 during school hours in the computer laboratories of the participating schools. To protect participants’ privacy and improve data quality, the research team conducted a preliminary inspection of the facilities before administration. When computers were positioned too close to one another, smaller testing sessions were organized so that students could complete the questionnaire at an adequate distance from one another.

The survey was administered online through the Alchemer platform, licensed to the University of Campania “Luigi Vanvitelli”, and accessed via a direct link provided on each computer. No data were stored on school computers, as all responses were recorded securely online. Within the same survey session, participants were randomly assigned to one of the four vignette conditions and completed the study materials individually and anonymously. Participation was voluntary, and students were informed that they could withdraw at any time without penalty.

The study was conducted in accordance with the Declaration of Helsinki. Written informed consent was obtained from parents or legal guardians prior to data collection, and assent was obtained from all adolescent participants. Teachers and families were informed in advance about the aims and procedures of the study, and participants were assured of the confidentiality of their responses.

### 2.4. Variables

Dependent variables. The dependent variables were the 14 adapted peer-response items administered after the vignette task. Each item captured a possible response to a peer disclosure of TDV and had three response categories: “I do not know”, “Yes”, and “No”. In all adult- and authority-oriented items, the response referred to an action that the disclosure recipient would take, or would encourage the disclosed friend to take, on behalf of that friend (e.g., involving a parent, school professional, principal, or law enforcement), rather than to the respondent seeking help for themselves.

Independent variable. The primary independent variable was vignette condition, with four levels: (1) heterosexual male-perpetrator/female-victim, (2) heterosexual female-perpetrator/male-victim, (3) male same-sex male-perpetrator/male-victim, and (4) female same-sex female-perpetrator/female-victim.

Moderator and covariate. Sex assigned at birth was examined as a moderator of condition effects, and age was included as a covariate in the multinomial logistic regression models.

### 2.5. Statistical Analysis

Descriptive analyses were first conducted to summarize the frequency distribution of responses to the 14 peer-response items in the total sample. Each item was scored using three response categories (“I don’t know”, “Yes”, and “No”), and descriptive percentages were computed for each category. Because “Yes” was used as the reference response category, each multinomial model estimated two non-reference contrasts for every item: “I do not know” versus “Yes” and “No” versus “Yes.” Full parameter estimates for both contrasts are reported in the Supplementary Materials (Table S3), whereas the main text focuses on omnibus model tests and on the contrast(s) that accounted for significant interaction effects.

To examine whether peer responses differed across vignette conditions, chi-square tests of independence were conducted separately for participants assigned male at birth and participants assigned female at birth. For outcomes showing significant omnibus associations, adjusted standardized residuals were inspected to identify the cells contributing most strongly to the observed association, with values of approximately |1.96| or greater treated as meaningful departures from expectation. To formally test whether the association between vignette condition and response category varied as a function of sex assigned at birth, multinomial logistic regression models were estimated for each response item. For each peer response item, multinomial logistic regression models were estimated with response category as the dependent variable, vignette condition and sex assigned at birth were entered as categorical predictors, and age was included as a covariate. Sex assigned at birth was coded 0 = male and 1 = female. Condition 4 (female same-sex couple) was used as the reference category. The Sex × Condition interaction term was included to test moderation. For all analyses, statistical significance was set at *p* < 0.05. Given the exploratory nature of the analyses, no correction for multiple testing was applied. Where model estimation was affected by sparse data or singularities in the Hessian matrix, results were treated as unstable and were not interpreted further. All analyses were conducted using IBM SPSS Statistics for Windows, version 30.0.0 (IBM Corp., 2021).

To further evaluate the internal organization of the 14 adapted response items, we examined Spearman inter-item associations and conducted an exploratory principal component analysis (PCA) with Varimax rotation on recoded item responses (0 = No, 1 = I do not know, 2 = Yes). The resulting factor loadings, communalities, and internal consistency estimates are reported in the Results section, whereas the full inter-item correlation matrix is provided in Supplementary Table S1. In addition, because the multinomial models required estimation across eight sex-by-condition subpopulations (the four vignette conditions within each sex-assigned-at-birth group), we also inspected subgroup-level response frequencies to identify low-frequency cells that could compromise model stability; a summary of these distributions is reported in Supplementary Table S2.

## 3. Results

### 3.1. Descriptive Frequencies of Peer Response Options

At the descriptive level, adolescents’ intended responses to peer disclosure in the overall sample were predominantly supportive and relational rather than formal or institutional. The most frequently endorsed strategies were listening to the friend (85.8%), helping the friend decide what to do after hearing what had happened (85.5%), encouraging the friend to talk to a trusted adult (78.3%), reassuring the friend that they were not to blame (76.2%), encouraging the friend to end the relationship (75.4%), and encouraging the friend to talk to a parent/guardian (74.2%). Talking to another friend (62.1%), talking to a trusted classmate (59.1%), and speaking directly to the friend’s partner (50.7%) were also relatively common. By contrast, institutional responses were less often endorsed: only 16.2% reported that they would contact law enforcement, 23.7% that they would talk to the school principal, and 32.1% that they would speak to the school psychologist; 15.1% indicated that they would do nothing. Overall, this pattern suggests a clear preference for informal, supportive, and accompaniment-based strategies over immediate recourse to formal authorities. Descriptive frequencies of peer responses are reported in Table 1.

### 3.2. Inter-Item Associations, Exploratory Dimensionality, and Cell Distribution Checks

Inter-item associations among the 14 adapted response items were heterogeneous, with Spearman coefficients ranging from −0.209 to 0.646, indicating that the items did not behave as interchangeable indicators of a single construct (see Supplementary Table S1). This approach was informed by Morrison et al. (2023), who first inspected inter-item correlations among their response items, then used PCA with Varimax rotation to identify broader response groupings, and finally retained weakly integrated but theoretically relevant items as individual indicators rather than forcing them into composite scores. An exploratory PCA with Varimax rotation supported a partially structured three-factor solution. The matrix was suitable for factor analysis (KMO = 0.789; Bartlett’s test of sphericity, χ^2^ (91) = 1733.317, *p* < 0.001), and the retained three-factor solution accounted for 46.401% of the total variance. As shown in Table 2, the first factor captured supportive/encouraging responses, the second captured adult-/institution-oriented responses, and the third captured peer-oriented responses. Internal consistency (Table 3) was acceptable for the supportive/encouraging factor (α = 0.756) and modest but acceptable for the adult-/institution-oriented factor (α = 0.681), whereas the peer-oriented factor showed weak internal consistency (α = 0.466). Two items (i.e., “I would do nothing” and “I would talk directly to my friend’s partner”) showed poor communalities (0.117 and 0.089, respectively) and did not integrate well into the common factor structure; consistent with the item-level logic also adopted by Morrison et al. (2023) for weakly integrated but conceptually meaningful responses, they were therefore retained as theoretically distinct single items. Finally, inspection of response frequencies across the eight sex-by-condition subpopulations showed that sparse cells were concentrated primarily in highly endorsed supportive items, especially helping the friend decide what to do after hearing what happened and listening to the friend, for which the “No” category was rare or absent in some subgroups (see Supplementary Table S2). These distributions help explain the convergence problems observed in the corresponding multinomial models and support the decision not to interpret unstable estimates.

### 3.3. Stratified Chi-Square Analyses by Sex Assigned at Birth

To examine condition effects more closely, chi-square analyses were conducted separately by sex assigned at birth. As outlined in the study aims, we expected any condition effects to be most visible for responses involving escalation beyond the peer dyad, such as involving adults, authorities, or recommending relationship termination. For outcomes showing significant omnibus associations, follow-up interpretation relied on the pattern of adjusted standardized residuals to identify the cells that contributed most to the observed associations.

Among participants assigned male at birth, condition differences emerged for three response items. First, willingness to talk to the principal varied by condition, χ^2^ (6, N = 283) = 19.78, *p* = 0.003, with the heterosexual female-perpetrator/male-victim condition showing fewer Yes responses and more No responses than expected, whereas the female same-sex female-perpetrator/female-victim condition showed more Yes responses than expected. Second, willingness to talk to the friend’s parent(s) or guardian also differed by condition, χ^2^ (6, N = 283) = 27.19, *p* < 0.001. Again, the clearest contribution came from the heterosexual female-perpetrator/male-victim condition, which showed fewer Yes responses and more No responses than expected, whereas the heterosexual male-perpetrator/female-victim condition showed fewer No responses than expected. Third, condition differences emerged for encouraging the friend to end the relationship, χ^2^ (6, N = 283) = 25.28, *p* < 0.001. In the heterosexual male-perpetrator/female-victim condition, participants showed fewer I don’t know responses and more Yes responses than expected, whereas the heterosexual female-perpetrator/male-victim condition showed fewer Yes responses and more No responses than expected. In all three cases, the same-sex conditions tended to occupy a more intermediate position. These significant condition effects are summarized in Table 4.

Among participants assigned female at birth, condition differences also emerged for three strategies. Willingness to talk to the friend’s parent(s) or guardian varied significantly across conditions, χ^2^ (6, N = 372) = 18.68, *p* = 0.005, with the heterosexual female-perpetrator/male-victim condition showing fewer Yes responses and more No responses than expected, whereas the female same-sex female-perpetrator/female-victim condition showed fewer “No” responses than expected. Condition was also associated with willingness to contact law enforcement, χ^2^ (6, N = 372) = 14.74, *p* = 0.022: the heterosexual male-perpetrator/female-victim condition showed more Yes responses than expected, whereas the heterosexual female-perpetrator/male-victim condition showed more No responses than expected. Finally, condition differences emerged for encouraging the friend to talk to a parent or guardian, χ^2^ (6, N = 372) = 12.94, *p* = 0.044. This effect was weaker and more localized, with the male same-sex male-perpetrator/male-victim condition showing more I don’t know responses than expected, and the heterosexual female-perpetrator/male-victim condition showing more No responses than expected. These significant condition effects are also summarized in Table 4.

Taken together, the stratified analyses suggest that condition effects were selective rather than pervasive and concentrated primarily on responses involving escalation to adults or authorities and, among male-assigned participants, recommendation to end the relationship. Across both sex-assigned-at-birth subgroups, the heterosexual female-perpetrator/male-victim condition was the scenario most consistently associated with reduced intervention-oriented responding and/or greater uncertainty. By contrast, the heterosexual male-perpetrator/female-victim condition more often elicited active intervention, particularly among male-assigned participants for involving authority figures and recommending relationship termination, and among female-assigned participants for contacting law enforcement. The two same-sex conditions generally showed a more intermediate profile. Significant stratified chi-square associations and the corresponding residual patterns are summarized in Table 4.

### 3.4. Multinomial Logistic Regression Models

To formally test whether the association between vignette condition and peer response categories varied as a function of sex assigned at birth, multinomial logistic regression models were estimated for each response item. In each model, the three response categories were treated as nominal, vignette condition and sex assigned at birth were entered as categorical predictors, age was included as a covariate, and the Sex × Condition interaction was tested. Sex assigned at birth was coded 0 = male and 1 = female; condition was coded with Condition 4 (female same-sex female-perpetrator/female-victim) as the reference category.

Across the multinomial models, omnibus Sex × Condition interactions were selective rather than pervasive after adjustment for age. Two models—helping the friend decide what to do after hearing what happened and listening to the friend—showed singularities in the Hessian matrix and were therefore not interpreted further. Across the remaining models, significant Sex × Condition interactions emerged only for talking to a trusted classmate, χ^2^ (6) = 12.78, *p* = 0.047, and talking to the friend’s parent(s) or guardian, χ^2^ (6) = 14.30, *p* = 0.026. For talking to the school principal, the interaction approached but did not reach conventional significance, χ^2^ (6) = 12.37, *p* = 0.054. All other interactions were non-significant (*ps* ≥ 0.137). In line with the exploratory nature of the analyses and the sparse distributions observed for some outcomes, individual parameter estimates are described only for models supported by a significant omnibus interaction test; isolated coefficients from non-significant omnibus interactions are not emphasized.

For talking to a trusted classmate, the significant interaction was driven primarily by the No-versus-Yes comparison: relative to the female same-sex female-perpetrator/female-victim condition, the heterosexual female-perpetrator/male-victim condition was associated with higher odds of rejecting this strategy rather than endorsing it among males relative to females (B = 1.499, OR = 4.478, 95% CI [1.331, 15.068], *p* = 0.015). No significant interaction terms emerged in the “I do not know” versus “Yes” contrast. For talking to the friend’s parent(s) or guardian, the significant interaction was driven mainly by the I-don’t-know-versus-Yes comparison, indicating sex-specific differences in uncertainty about involving parents/guardians rather than in endorsement per se. Specifically, the heterosexual female-perpetrator/male-victim condition was associated with higher odds of responding “I don’t know” rather than “Yes” among males relative to females (B = 1.437, OR = 4.208, 95% CI [1.323, 13.381], *p* = 0.015). By contrast, in the “No” versus “Yes” comparison, no interaction terms reached significance, suggesting that the omnibus interaction was driven mainly by sex-specific differences in uncertainty rather than in rejection of the response. For talking to the school principal, the omnibus interaction approached but did not reach conventional significance. Although some individual coefficients in this model reached significance, they are not interpreted as conclusive evidence of moderation because the overall Sex × Condition interaction was not statistically significant, χ^2^ (6) = 12.37, *p* = 0.054. Full parameter estimates for these models are reported in the Supplementary Materials (Table S3).

Age effects were generally limited. Age was significantly associated with response category only for talking to the school principal, χ^2^ (2) = 14.95, *p* < 0.001, such that older participants were more likely to respond either “I don’t know” rather than “Yes” (B = 0.218, OR = 1.244, 95% CI [1.085, 1.426], *p* = 0.002) or “No” rather than “Yes” (B = 0.264, OR = 1.302, 95% CI [1.130, 1.499], *p* < 0.001). An omnibus age effect also emerged for talking to the friend’s parent(s) or guardian, χ^2^ (2) = 6.65, *p* = 0.036, whereas age was otherwise non-significant or marginal across the remaining models. The omnibus results of the multinomial logistic regression models are summarized in Table 5.

## 4. Discussion

The present study extends the literature on adolescents’ responses to peer disclosures of dating violence by showing that response intentions are shaped not only by a general preference for supportive responses, but also by the relational configuration through which the episode is made intelligible. This is important because adolescents’ first responses as peer recipients of disclosure may influence whether the disclosing victim feels believed, supported, and safe enough to seek additional help, thereby affecting not only immediate coping but also broader mental health and adjustment trajectories (Fernet et al., 2021; Muñoz-Fernández et al., 2026). Across the full sample, adolescents predominantly endorsed informal, relational, and accompaniment-based strategies, such as listening to the friend, helping them decide what to do, reassuring them that they were not to blame, or encouraging them to talk to trusted others. By contrast, institutional responses such as contacting law enforcement, talking to the principal, or talking to the school psychologist were endorsed much less often, suggesting a relative preference for informal and relational responses over institutional escalation in the context of a peer disclosure scenario. This pattern is broadly consistent with prior findings showing that adolescents who themselves experience dating violence often rely more on informal than formal sources of support (Ashley & Foshee, 2005; Fernet et al., 2021). Although those studies concern help-seeking by adolescents experiencing TDV rather than responses by disclosure recipients, they help contextualize why listening, reassurance, advice, and encouragement to talk to trusted others were so frequently endorsed in the present study. More specifically, qualitative evidence indicates that adolescents experiencing dating violence often seek informal support not only for advice, but also to vent emotions, obtain an external point of view, feel listened to and comforted, and receive validation for their interpretation of the situation (Fernet et al., 2021). The prominence of responses such as listening to the friend, helping them decide what to do, reassuring them, and encouraging them to talk to trusted others is therefore highly consistent with the kinds of support that disclosing adolescents describe as useful. At the same time, the present data do not identify why institutional responses were less frequently endorsed; rather, they indicate that participants more readily imagined providing first-line interpersonal support than immediately involving formal authorities or institutional channels (Muñoz-Fernández et al., 2026).

The additional psychometric checks suggest that the adapted items are best understood as a heterogeneous response set with partial clustering, rather than as a single unified scale. Two relatively coherent families of responses emerged: supportive/encouraging and adult-/institution-oriented; in contrast, peer-oriented responses were more weakly integrated, and two items (“I would do nothing” and “I would talk directly to my friend’s partner”) behaved as conceptually distinct ctions. This pattern supports a mixed interpretive framework: the response items share some internal organization, but they are not interchangeable indicators of a single latent construct. At the same time, the sparse distribution of “No” responses for highly endorsed supportive items, especially helping the friend decide what to do and listening to the friend, indicates that some multinomial models were likely overparameterized relative to the available subgroup cell frequencies. Accordingly, the interaction findings should be regarded as selective and provisional, and the unstable models should not be interpreted as robust evidence of moderation.

At the same time, condition effects were selective rather than pervasive. Differences did not emerge across all response items. Instead, they were concentrated primarily in items that required participants to move beyond first-line peer support and consider escalation, such as talking to the school principal, talking to the friend’s parent(s) or guardian, contacting law enforcement, or recommending that the friend end the relationship. In practical terms, this means that adolescents responded relatively similarly across vignette conditions when imagining supportive first responses such as listening, reassuring, or helping the friend think about what to do, whereas they became more condition-sensitive when deciding whether the disclosed situation warranted involvement of adults, authorities, or a clear recommendation to leave the relationship. These differences matter because they shape whether the disclosing friend is likely to be encouraged toward broader support beyond the immediate peer interaction, including parents, school professionals, or institutional resources. In this respect, the present findings complement Morrison et al. (2023): whereas their study showed that the type of dating violence influenced adolescents’ reactions, the present study indicates that the gendered configuration of the relationship itself also matters.

Most notably, across both sex-assigned-at-birth subgroups, the heterosexual female-perpetrator/male-victim condition was the scenario most consistently associated with reduced intervention-oriented responding and/or greater uncertainty, whereas the heterosexual male-perpetrator/female-victim condition more often elicited active intervention, particularly with respect to involving parents, authorities, or recommending relationship termination. These condition differences should not be interpreted as direct evidence of specific sociocultural mechanisms, because constructs such as gender norms, heteronormativity, minority stress, or perceived social legibility were not directly measured in the present study. Rather, the findings are consistent with the possibility that some scenarios were more readily recognized as warranting intervention than others. These processes remain plausible explanatory hypotheses for future research, but they cannot be tested directly in the current dataset. This interpretation remains broadly compatible with prior work on TDV risk and recognition among sexual and gender minority youth (Sulla et al., 2025).

These findings may be further understood by considering not only the recognizability of the violent scenario, but also the recognizability of the support system to which adolescents might turn. If some forms of teen dating violence are less readily interpreted as violence, adolescents may be more hesitant to activate adult or institutional responses; this hesitation may be amplified when school-based support is itself perceived as uneven, implicit, or insufficiently visible. Recent school-based evidence shows that students often perceive adult involvement as less consistent than teachers report in addressing issues such as respect, consent, jealousy, control, and affective relationships (Lavanga et al., 2026). Accordingly, adolescents may be more likely to imagine involving adults when both the violent episode and the available adult support are perceived as recognizable and credible within everyday school life.

A further important finding is that sex assigned at birth did not operate as a pervasive moderator of peer responses to disclosure. Although the stratified chi-square analyses showed some condition-specific differences, the multinomial models indicated that only a small number of Sex × Condition interactions remained significant once age was taken into account, and two models proved too unstable to interpret because of sparse data. This suggests that respondent sex assigned at birth shapes response intentions only selectively, rather than systematically reorganizing the whole pattern of condition effects. Because the present study assessed adolescents’ intended responses to a friend’s disclosure (not their own help-seeking if they were personally experiencing TDV) these findings should not be interpreted as evidence that girls and boys necessarily seek help differently for themselves. Rather, they suggest that male- and female-assigned participants differed only modestly, and only in selected scenarios, in how they imagined responding to a disclosed episode. More broadly, the findings fit with a socio-ecological view of adolescent responses to violence: responses to disclosure appear to be embedded in a wider ecology of peer norms, social recognition of violence, and school-based messages about whether involving adults is seen as credible, appropriate, or necessary. Research on bullying has similarly shown that many adolescents do not seek help even when help-seeking is the strategy adults most strongly recommend, and that informal support is often preferred over formal intervention; importantly, when adult responses are perceived as passive, disengaged, or contradictory, students may experience reduced safety, lower trust, and weaker school belonging, all of which are relevant to mental health and school adjustment (Muñoz-Fernández et al., 2026).

### 4.1. Practical Implications

From an applied perspective, these findings indicate that prevention efforts should move beyond generic messages about supporting a friend and should explicitly address how adolescents interpret different configurations of dating violence. School-based programs should include scenarios involving boys as victims, girls as perpetrators, and same-sex couples, so that adolescents can learn to recognize harm even when it does not fit dominant heteronormative expectations. Making these less recognized scenarios visible may help reduce uncertainty, challenge minimization, and improve willingness to intervene.

The findings also highlight the importance of strengthening peer-response efficacy, not only as a violence-prevention resource but also as a potential mental health protection mechanism. Because adolescents appear especially likely to offer informal support before involving formal adults or institutions, programs should teach concrete peer-support skills: how to listen non-judgmentally, how to validate a friend’s experience, how to avoid victim blaming, how to provide an external perspective without being dismissive, how to encourage help-seeking without taking control away from the victim, and how to recognize when the situation requires escalation to a trusted adult or professional. These components align closely with the kinds of support adolescents themselves describe as useful when facing romantic difficulties or dating violence (Fernet et al., 2021). In this sense, peer education and bystander-oriented interventions may be especially valuable. At the same time, future research should clarify whether adolescents’ willingness to recommend adult-oriented help to a peer corresponds to the help-seeking choices they would anticipate making for themselves, since prior studies suggest that adolescents experiencing dating violence often rely more on informal than formal sources of support (Ashley & Foshee, 2005; Fernet et al., 2019).

These implications are also consistent with broader school-based prevention research indicating that effective dating and relationship violence and gender-based violence programs may need to operate at multiple levels, including students, school personnel, family members, and the wider school environment (Rizzo et al., 2023), and with evidence that bystander-oriented school programs can strengthen adolescents’ readiness to intervene in violence-related situations (Jouriles et al., 2019).

At the school level, teacher training and school mental health services should also take into account that adolescents may be reluctant to activate institutional channels, particularly when the situation appears ambiguous or socially less legible. Consistent, visible, and inclusive adult responses are likely to matter not only for disclosure and referral, but also for students’ perceived safety, trust in school, and emotional adjustment. Schools could therefore benefit from creating multiple, low-threshold pathways for disclosure, including trained teachers, counselors, psychologists, and anonymous or semi-anonymous reporting options. Interventions should not assume that adolescents will automatically interpret all forms of TDV as equally serious; rather, they should help students distinguish between situations that call primarily for emotional support and those that warrant adult, clinical, or institutional involvement. Overall, a more inclusive and developmentally tailored prevention framework may improve adolescents’ capacity to identify TDV, respond supportively to peers, and mobilize appropriate forms of help across a broader range of relationship contexts, with likely benefits for both violence prevention and youth mental health.

### 4.2. Limitations

Several limitations should be acknowledged. First, the study relied on a convenience sample drawn from secondary schools in Southern Italy and may therefore not be representative of the broader adolescent population. Second, all measures were based on self-report and, despite the guarantee of anonymity, responses may still have been influenced by social desirability or other reporting biases. This issue may be especially relevant in vignette-based research, where participants may indicate what they believe would be the most appropriate response to recommend to someone else, rather than what they would actually do if personally involved in a comparable situation. Indeed, qualitative research on dating violence help-seeking suggests that actual disclosure decisions are shaped by additional barriers—including discomfort with sharing intimate information, fear of judgment, fear of appearing vulnerable, concerns about others’ perceptions of the partner, and the possibility of not having anyone truly available or supportive to turn to (Fernet et al., 2021). Relatedly, the present design does not allow us to determine whether the same participants would endorse similar help-seeking choices if they themselves were the victim. This distinction is important, given previous evidence suggesting that adolescents experiencing dating violence often do not seek help at all and, when they do, tend to rely more on informal than formal sources of support (Ashley & Foshee, 2005; Fernet et al., 2019).

An additional limitation concerns the internal organization of the 14 adapted response items. Exploratory analyses indicated that these items did not function as a single homogeneous construct, but rather as a partially structured set of responses with varying degrees of coherence. Although this supports an item-level interpretation for theoretically distinct responses, it also suggests that the response battery should be treated cautiously as a heterogeneous set rather than as a unified scale.

In addition, as with all vignette-based studies, the scenarios necessarily simplified complex relational situations and may themselves reflect implicit assumptions embedded in their construction. Finally, the cross-sectional nature of the design precludes causal inference. In addition, several response items were highly skewed toward endorsement, which produced sparse “No” categories in some sex-by-condition subpopulations; this contributed to instability in two multinomial models and warrants caution in interpreting interaction effects more generally. Moreover, the sociocultural processes invoked in the discussion—such as social recognizability, gender norms, or heteronormative assumptions—were not directly measured in the present study and should therefore be treated as plausible explanatory hypotheses rather than as directly tested mechanisms.

Future studies should directly compare adolescents’ third-person recommendations in vignette-based situations with first-person anticipated or actual help-seeking if they themselves were the victim, use larger and more diverse samples, and adopt longitudinal designs to clarify how response intentions relate to later disclosure, bystander behavior, and the relational barriers and facilitators that shape informal help-seeking in real contexts. It would also be important to include direct measures of the sociocultural processes discussed here, as well as response formats that permit more stable modelling of highly endorsed categories.

### 4.3. Conclusions

Adolescents in this study generally endorsed supportive responses to peer disclosure of TDV, but these intentions were not evenly distributed across relationship configurations. Responses involving escalation to adults or institutions were more sensitive to the social recognizability of the vignette scenario than first-line supportive actions. Overall, the findings suggest that peer responses to disclosure are shaped both by prosocial intentions and by how legible the violent situation appears.

## Figures and Tables

**Table 1:** Descriptive frequencies of peer response options in the total sample (N = 655).

Peer Response	I Don’t Know (%)	Yes (%)	No (%)
I would do nothing	23.2	15.1	61.7
I would talk to the school principal	39.8	23.7	36.5
I would talk to a trusted classmate	22.9	59.1	18.0
I would talk to the school psychologist	32.1	32.1	35.9
I would talk directly to my friend’s partner	30.1	50.7	19.2
I would talk to my friend’s parent(s)/guardian	28.7	43.7	27.6
I would contact law enforcement	31.8	16.2	52.1
I would reassure my friend that they are not to blame	15.0	76.2	8.9
I would help my friend decide what to do after hearing what happened	11.3	85.5	3.2
I would listen to the friend	10.8	85.8	3.4
I would encourage my friend to talk to a trusted adult	15.7	78.3	6.0
I would encourage my friend to talk to a parent/guardian	17.4	74.2	8.4
I would encourage my friend to end the relationship	17.9	75.4	6.7
I would encourage my friend to talk to another friend	26.0	62.1	11.9

**Table 2:** Exploratory principal component analysis and internal consistency of the adapted response items.

Item	Factor 1 Supportive/Encouraging	Factor 2 Adult-/Institution-Oriented	Factor 3 Peer-Oriented	Communality
I would help my friend decide what to do after hearing what happened (FR9)	0.753			0.570
I would listen to the friend (FR10)	0.701			0.518
I would encourage my friend to talk to a trusted adult (FR11)	0.664			0.549
I would reassure my friend that they are not to blame (FR8)	0.617			0.383
I would encourage my friend to talk to a parent/guardian (FR12)	0.593	0.363		0.507
I would encourage my friend to end the relationship (FR13)	0.527	0.467		0.460
I would do nothing (FR1)	−0.333			0.117
I would talk to my friend’s parent(s)/guardian (FR6)		0.722		0.567
I would talk to the school principal (FR2)		0.717		0.531
I would contact law enforcement (FR7)		0.678		0.473
I would talk to the school psychologist (FR4)		0.661		0.453
I would talk to a trusted classmate (FR3)			0.830	0.704
I would encourage my friend to talk to another friend (FR14)			0.712	0.573
I would talk directly to my friend’s partner (FR5)				0.089

Note. Only loadings with absolute value ≥ 0.30 are displayed. PCA was conducted on recoded responses (0 = No, 1 = I do not know, 2 = Yes) using Varimax rotation. The three-factor solution accounted for 46.401% of total variance. FR1 and FR5 showed poor communalities and were retained as theoretically distinct single items rather than included in composite interpretations.

**Table 3:** Factor-level indices.

Factor	Items	Cronbach’s α
Supportive/encouraging responses	FR8, FR9, FR10, FR11, FR12, FR13	0.756
Adult-/institution-oriented responses	FR2, FR4, FR6, FR7	0.681
Peer-oriented responses	FR3, FR14	0.466

**Table 4:** Stratified Chi-Square Analyses by Sex Assigned at Birth: Significant Condition Effects and Main Residual Patterns.

Peer Response	Sex-Assigned-At-Birth Group	χ^2^ (df)	*p*	Main Residual Pattern
Talk to the school principal	Male-assigned	19.78 (6)	0.003	In the heterosexual female-perpetrator/male-victim condition, fewer Yes and more No responses than expected; in the female same-sex condition, more Yes responses than expected.
Talk to the friend’s parent(s)/guardian	Male-assigned	27.19 (6)	<0.001	In the heterosexual female-perpetrator/male-victim condition, fewer Yes and more No responses than expected; in the heterosexual male-perpetrator/female-victim condition, fewer No responses than expected.
Encourage the friend to end the relationship	Male-assigned	25.28 (6)	<0.001	In the heterosexual male-perpetrator/female-victim condition, fewer I don’t know and more Yes responses than expected; in the heterosexual female-perpetrator/male-victim condition, fewer Yes and more No responses than expected.
Talk to the friend’s parent(s)/guardian	Female-assigned	18.68 (6)	0.005	In the heterosexual female-perpetrator/male-victim condition, fewer Yes and more No responses than expected; in the female same-sex condition, fewer No responses than expected.
Contact law enforcement	Female-assigned	14.74 (6)	0.022	In the heterosexual male-perpetrator/female-victim condition, more Yes responses than expected; in the heterosexual female-perpetrator/male-victim condition, more No responses than expected.
Encourage the friend to talk to a parent/guardian	Female-assigned	12.94 (6)	0.044	In the male same-sex condition, more I don’t know responses than expected; in the heterosexual female-perpetrator/male-victim condition, more No responses than expected.

Note. Only significant omnibus χ^2^ results are shown. Follow-up interpretation was based on adjusted standardized residuals, with values of approximately |1.96| or greater treated as meaningful departures from expectation.

**Table 5:** Summary of Multinomial Logistic Regression Models Testing Sex × Condition Effects on Peer Response Categories. Multinomial logistic models used “Yes” as the reference response category and Condition 4 (female same-sex couple) as the reference condition; age was included as a covariate. Omnibus tests are based on likelihood-ratio chi-square statistics.

Peer Response	Overall Model χ^2^ (df)	*p*	Age χ^2^ (df)	*p*	Sex × Condition χ^2^ (df)	*p*	Interpretation	Primary Non-Reference Contrast(s) Interpreted
I would do nothing	24.87 (16)	0.072	0.07 (2)	0.968	8.57 (6)	0.199	Interaction non-significant	No interaction interpreted
Talk to the school principal	48.15 (16)	<0.001	14.95 (2)	<0.001	12.37 (6)	0.054	Trend-level interaction	Trend-level omnibus; coefficients not emphasized
Talk to a trusted classmate	17.98 (16)	0.325	0.48 (2)	0.786	12.78 (6)	0.047	Interaction significant	No vs. Yes
Talk to the school psychologist	11.98 (16)	0.745	1.22 (2)	0.543	3.17 (6)	0.787	Interaction non-significant	No interaction interpreted
Talk directly to the friend’s partner	17.17 (16)	0.375	2.42 (2)	0.299	6.48 (6)	0.372	Interaction non-significant	No interaction interpreted
Talk to the friend’s parent(s)/guardian	61.95 (16)	<0.001	6.65 (2)	0.036	14.30 (6)	0.026	Interaction significant	I do not know vs Yes
Contact law enforcement	27.59 (16)	0.035	2.58 (2)	0.275	9.70 (6)	0.138	Interaction non-significant	No interaction interpreted
Reassure the friend that they are not to blame	64.21 (16)	<0.001	5.53 (2)	0.063	4.73 (6)	0.579	Interaction non-significant	No interaction interpreted
Help the friend decide what to do after hearing what happened	36.94 (16)	0.002	3.19 (2)	0.203	9.71 (6)	0.137	Model unstable; not interpreted	Model unstable
Listen to the friend	43.29 (16)	<0.001	0.48 (2)	0.787	5.86 (6)	0.439	Model unstable; not interpreted	Model unstable
Encourage the friend to talk to a trusted adult	54.78 (16)	<0.001	3.60 (2)	0.165	1.83 (6)	0.935	Interaction non-significant	No interaction interpreted
Encourage the friend to talk to a parent/guardian	48.04 (16)	<0.001	3.05 (2)	0.218	1.31 (6)	0.971	Interaction non-significant	No interaction interpreted
Encourage the friend to end the relationship	66.43 (16)	<0.001	5.82 (2)	0.054	3.73 (6)	0.714	Interaction non-significant	No interaction interpreted
Encourage the friend to talk to another friend	25.29 (16)	0.065	3.03 (2)	0.220	3.78 (6)	0.707	Interaction non-significant	No interaction interpreted

## Data Availability

The data are not publicly available due to ethical and privacy restrictions involving minor participants but may be available from the corresponding author upon reasonable request and subject to institutional approval.

## Supplementary Materials

The following supporting information can be downloaded at https://www.mdpi.com/article/10.3390/bs16071043/s1. Table S1. Spearman inter-item correlations among the 14 adapted response items (N = 655). Table S2. Low-frequency response-category counts across sex × condition subpopulations. Table S3. Full parameter estimates of the multinomial logistic regression models. Text S1. English translation of the vignette content. Text S2. Fourteen adapted response items: Italian wording and English counterparts.
