# Supplementary material for: Adolescents’ Responses to Peer Disclosure of Teen Dating Violence: Relationship Configuration, Response Intentions, and Protective Adult Support
Source: Behav Sci (Basel). 2026 Jun 23;16(7):1043. doi: 10.3390/bs16071043 (PMC13404815; doi:10.3390/bs16071043)
Supplement: Supplementary file 1 [file behavsci-16-01043-s001.zip › behavsci-4318228-supplementary.pdf]

Supplementary materials

S1. Table S1

Supplementary Table S1

Spearman inter-item correlations among the 14 adapted response items (N = 655)

Item	FR1	FR2	FR3	FR4	FR5	FR6	FR7	FR8	FR9	FR10	FR11	FR12	FR13	FR14
**FR1**	1.000													
**FR2**	.022	1.000												
**FR3**	-.068	.101**	1.000											
**FR4**	-.048	.403**	.115**	1.000										
**FR5**	-.106**	.001	.031	.017	1.000									
**FR6**	-.077*	.373**	.057	.313**	.062	1.000								
**FR7**	.020	.334**	.013	.312**	-.055	.379**	1.000							
**FR8**	-.204**	-.044	.120**	.044	.126**	.109**	-.030	1.000						
**FR9**	-.192**	-.005	.138**	.095*	.189**	.106**	-.064	.365**	1.000					
**FR10**	-.192**	-.040	.197**	.032	.131**	.123**	-.036	.405**	.489**	1.000				
**FR11**	-.160**	.138**	.235**	.170**	.053	.302**	.123**	.283**	.381**	.357**	1.000			
**FR12**	-.095*	.161**	.161**	.192**	.034	.373**	.136**	.233**	.366**	.297**	.646**	1.000		
**FR13**	-.209**	.033	.091*	.054	.117**	.180**	.050	.338**	.402**	.343**	.313**	.305**	1.000	

Item	FR1	FR2	FR3	FR4	FR5	FR6	FR7	FR8	FR9	FR10	FR11	FR12	FR13	FR14
**FR14**	-.102**	.001	.316**	.039	.153**	.026	-.022	.192**	.207**	.255**	.262**	.232**	.209**	1.000

Note. Values are Spearman's rho correlations among the 14 adapted response items, computed on recoded responses (0 = No, 1 = I do not know, 2 = Yes). Item labels were as follows: FR1 = I would do nothing; FR2 = I would talk to the school principal; FR3 = I would talk to a trusted classmate; FR4 = I would talk to the school psychologist; FR5 = I would talk directly to my friend's partner; FR6 = I would talk to my friend's parent(s)/guardian; FR7 = I would contact law enforcement; FR8 = I would reassure my friend that they are not to blame; FR9 = I would help my friend decide what to do after hearing what happened; FR10 = I would listen to the friend; FR11 = I would encourage my friend to talk to a trusted adult; FR12 = I would encourage my friend to talk to a parent/guardian; FR13 = I would encourage my friend to end the relationship; FR14 = I would encourage my friend to talk to another friend.
Correlations ranged from -.209 to .646.
** Correlation is significant at the .01 level (2-tailed)
* Correlation is significant at the .05 level (2-tailed)

S1. Table S2

Supplementary Table S2

Low-frequency response-category counts across sex × condition subpopulations

Item	Low-frequency category	Overall % of that category	M1	M2	M3	M4	F1	F2	F3	F4	Minimum	Zero cells?
**FR2 – Talk to the school principal**	Yes	23.7	27	5	12	23	25	20	24	19	5	No
**FR8 – Reassure the friend they are not to blame**	No	8.9	15	6	7	11	2	4	6	7	2	No
**FR9 – Help the friend decide what to do**	No	3.2	5	4	4	2	0	3	1	2	0	Yes
**FR10 – Listen to the friend**	No	3.4	4	3	4	3	0	5	2	1	0	Yes
**FR11 – Encourage the friend to talk to a trusted adult**	No	6.0	4	11	7	9	1	3	1	3	1	No

Item	Low-frequency category	Overall % of that category	M1	M2	M3	M4	F1	F2	F3	F4	Minimum	Zero cells?
**FR12 – Encourage the friend to talk to a parent/guardian**	No	8.4	9	12	6	8	2	10	4	4	2	No
**FR13 – Encourage the friend to end the relationship**	No	6.7	4	14	5	3	1	7	5	5	1	No

Note. Low-frequency categories were inspected across the eight sex × condition subpopulations to evaluate the stability of multinomial logistic models. The clearest sparse-data problems were concentrated in the No category for FR9 and FR10, where at least one subgroup had a cell count of 0. More moderate sparsity was also observed for the No category of FR11–FR13 and, to a lesser extent, FR8. These patterns were used to contextualize model instability and to justify non-interpretation of unstable multinomial models.
Subgroup sizes: M1 = Male_Cond1 (n = 87); M2 = Male_Cond2 (n = 70); M3 = Male_Cond3 (n = 58); M4 = Male_Cond4 (n = 68); F1 = Female_Cond1 (n = 79); F2 = Female_Cond2 (n = 101); F3 = Female_Cond3 (n = 95); F4 = Female_Cond4 (n = 97).

S1. Table S3

Supplementary Table S3 Full parameter estimates of the multinomial logistic regression models

Parameter estimates from multinomial logistic regression models showing significant or trend-level Sex × Condition effects. Sex assigned at birth was coded 0 = male and 1 = female. Condition 4 (female same-sex female-perpetrator/female-victim) served as the reference condition. Response category "Yes" served as the reference outcome category. Only coefficients relevant to significant or trend-level Sex × Condition effects are shown.

Section A. Talking to the school principal (trend-level interaction)

Outcome contrast	Predictor	B	OR	95% CI for OR	p
I don't know vs Yes	Age	0.218	1.244	[1.085, 1.426]	.002
I don't know vs Yes	Sex × Condition 2	2.196	8.992	[2.313, 34.953]	.002
I don't know vs Yes	Sex × Condition 3	1.398	4.046	[1.247, 13.127]	.020
No vs Yes	Age	0.264	1.302	[1.130, 1.499]	< .001
No vs Yes	Sex × Condition 2	1.645	5.180	[1.335, 20.101]	.017

Note. The omnibus Sex × Condition interaction was trend-level, $\chi^2(6) = 12.37$, $p = .054$, and therefore individual coefficients should be interpreted cautiously.

Section B. Talking to a trusted classmate (significant interaction)

Outcome contrast	Predictor	B	OR	95% CI for OR	p
No vs Yes	Sex × Condition 2	1.499	4.478	[1.331, 15.068]	.015

Note. The omnibus Sex × Condition interaction was significant, $\chi^2(6) = 12.78$, $p = .047$. This interaction was driven primarily by the No-versus-Yes contrast.

Section C. Talking to the friend’s parent(s) or guardian (significant interaction)

Outcome contrast	Predictor	B	OR	95% CI for OR	p
I don’t know vs Yes	Age	0.161	1.175	[1.036, 1.332]	.012
I don’t know vs Yes	Sex × Condition 2	1.437	4.208	[1.323, 13.381]	.015
No vs Yes	Sex (male vs female) in reference condition	0.909	2.482	[1.085, 5.678]	.031
No vs Yes	Condition 2 (vs Condition 4) among females	1.367	3.922	[1.826, 8.425]	< .001
No vs Yes	Condition 3 (vs Condition 4) among females	0.820	2.270	[1.047, 4.922]	.038

Note. The omnibus Sex × Condition interaction was significant, $\chi^2(6) = 14.30$, $p = .026$, and was driven primarily by the I-don’t-know-versus-Yes contrast. None of the interaction terms in the No-versus-Yes contrast reached significance.

Supplementary Text S1. English Translation of the Vignette Content

Note. Because the wording of the vignettes was identical across conditions, with only the composition of the couple and the order of the episodes varying, we report below the English translation of one condition only.

Introduction

Nancy is a 15-year-old girl in your class. She is a close friend of yours, gets good grades at school, and has been dating David, an older boy from another class, for about six months. During recess, she asks whether you would like to have a coffee with her after school to talk privately about something important. Nancy tells you that she has doubts about several incidents that happened in the past few days involving David.

Episode 1: Verbal-Emotional TDV

One incident happened at a pub. David and Nancy meet to join some friends at a pub. Nancy is wearing leggings, and David, looking annoyed, tells her that she could only

get away with wearing something like that if she decided to go to the gym. During dinner with their friends, Nancy takes some chips from David's plate, and he says loudly that, if she could, Nancy would even eat the waiter. Everyone laughs, and David then tells her that, if she keeps going like that, her leggings might explode. Feeling embarrassed, Nancy stops eating and starts looking at some posts on Instagram. David, now suspicious, looks at Nancy's phone to see whether she is messaging someone. After that, David spends the rest of the evening chatting and laughing with Nadia, a new girl in the group. As Nancy tells you the story, she seems confused about what happened and about what she should do.

Episode 2: Relational TDV/Threats

Another incident happened one evening while they were heading home. After spending the evening with friends, David and Nancy are riding home on David's moped when Nancy receives a WhatsApp message. Nancy looks at her phone and puts it in her pocket. At that point, David stops the moped and asks to see her phone. Nancy refuses, so David threatens to break her phone if she does not show it to him immediately. He then tells Nancy to get off the moped and says that he will leave her there and make her walk home. In the following days, Nancy finds out that David has spread rumors about her, saying that he left her there because he discovered that she had cheated on him. As Nancy tells you the story, she seems confused about what happened and about what she should do.

Episode 3: Physical TDV

Another incident happened in the afternoon. David starts asking Nancy why she does not reply to his messages when she is at school. Nancy says that she is very busy with classes while at school. David accuses Nancy of lying and cheating on him and tells her that, if she really loved him, she would spend much more time with him during the day. Nancy tries to hug David, telling him that she would never cheat on him, but David gets angry and pushes her. At one point, Nancy receives a friend request from Luca, a new male classmate with whom she is working on a project. David then explodes, takes Nancy's phone away from her, and throws it. As Nancy tells you the story, she seems confused about what happened and about what she should do.

Supplementary Text S2. Fourteen adapted response items: Italian wording and English counterparts

Supplementary Text S2. The English items were translated and adapted from the response-to-disclosure framework used by Morrison et al. (2023).

Adapted Italian Items	Original English Items
Non farei nulla	I would do nothing
Ne parlerei con il Dirigente Scolastico	I would talk to the school principal
Ne parlerei con un/una compagna di classe di cui mi fido	I would talk to a classmate I trust
Ne parlerei con lo/la psicologo/a scolastico	I would talk to the school psychologist
Parlerei direttamente al/alla fidanzato/a del mio/mia amico/amica	I would talk directly to my friend's boyfriend/girlfriend
Parlerei con il/i genitore/i e/o tutore del/della mio/mia amico/a	I would talk to my friend's parent(s) and/or guardian
Contatterei le forze dell'ordine	I would contact law enforcement
Rassicurerei il/la mio/mia amico/a che non ha alcuna colpa	I would reassure my friend that they are not to blame
Aiuterei il/la mio/mia amico/amica a decidere come comportarsi dopo aver ascoltato l'accaduto	I would help my friend decide what to do after hearing what happened
Ascolterei il/la mio/mia amico/amica	I would listen to my friend
Incoraggerei il/la mio/mia amico/amica a parlare con un adulto di cui si fida	I would encourage my friend to talk to a trusted adult
Incoraggerei il/la mio/mia amico/amica a parlare con un genitore/tutore	I would encourage my friend to talk to a parent/guardian
Incoraggerei il/la mio/mia amico/amica a porre fine alla relazione	I would encourage my friend to end the relationship
Incoraggerei il/la mio/mia amico/amica a parlare con un/una amico/amica	I would encourage my friend to talk to another friend
Altro (Specificare)	Other (please specify)
